# Assessment of Narrow Band Imaging Algorithm for Video Capsule Endoscopy Based on Decorrelated Color Space for Esophageal Cancer

**DOI:** 10.3390/cancers15194715

**Published:** 2023-09-25

**Authors:** Kai-Yao Yang, Yu-Jen Fang, Riya Karmakar, Arvind Mukundan, Yu-Ming Tsao, Chien-Wei Huang, Hsiang-Chen Wang

**Affiliations:** 1 Department of Medical Material Research, Kaohsiung Armed Forces General Hospital, 2, Zhongzheng 1st. Rd., Lingya District, Kaohsiung City 80284, Taiwan; yangkaiyao@gmail.com; 2 Department of Internal Medicine, National Taiwan University Hospital, Yun-Lin Branch, No. 579, Sec. 2, Yunlin Rd., Dou-Liu 64041, Taiwan; toby851072@gmail.com; 3 Department of Internal Medicine, National Taiwan University College, No. 1 Jen Ai Rd. Sec. 1, Taipei 10051, Taiwan; 4 Department of Mechanical Engineering, National Chung Cheng University, 168, University Rd., Min Hsiung, Chiayi 62102, Taiwan; karmakarriya345@gmail.com (R.K.); d09420003@ccu.edu.tw (A.M.); d09420002@ccu.edu.tw (Y.-M.T.); 5 Department of Nursing, Tajen University, 20, Weixin Rd., Yanpu Township, Pingtung 90741, Taiwan; 6 Department of Medical Research, Dalin Tzu Chi Hospital, Buddhist Tzu Chi Medical Foundation, No. 2, Minsheng Road, Dalin, Chiayi 62247, Taiwan; 7 Hitspectra Intelligent Technology Co., Ltd., 4F, No.2, Fuxing 4th Rd., Qianzhen District, Kaohsiung City 80661, Taiwan

**Keywords:** narrow band imaging, hyperspectral imaging, decorrelated color space, video capsule endoscopy, peak-signal-to-noise ratio, structural similarity index metric, entropy

## Abstract

**Simple Summary:**

Video capsule endoscopy (VCE) is a small, patient-friendly tool used for medical imaging, but it lacks narrow band imaging (NBI), which is crucial for detecting various cancers like esophageal cancer (EC). EC is hard to detect early since it often shows no symptoms, leading to a low 5-year survival rate. NBI enhances mucosal features for early cancer identification, but adding it to VCE isn’t feasible due to size constraints. This study successfully developed a method to convert traditional white light images (WLI) from VCE into NBI-like images for esophageal examination. The method performed well, with high similarity scores and improved image quality, offering a promising solution for better cancer detection.

**Abstract:**

Video capsule endoscopy (VCE) is increasingly used to decrease discomfort among patients owing to its small size. However, VCE has a major drawback of not having narrow band imaging (NBI) functionality. The current VCE has the traditional white light imaging (WLI) only, which has poor performance in the computer-aided detection (CAD) of different types of cancer compared to NBI. Specific cancers, such as esophageal cancer (EC), do not exhibit any early biomarkers, making their early detection difficult. In most cases, the symptoms are unnoticeable, and EC is diagnosed only in later stages, making its 5-year survival rate below 20% on average. NBI filters provide particular wavelengths that increase the contrast and enhance certain features of the mucosa, thereby enabling early identification of EC. However, VCE does not have a slot for NBI functionality because its size cannot be increased. Hence, NBI image conversion from WLI can presently only be achieved in post-processing. In this study, a complete arithmetic assessment of the decorrelated color space was conducted to generate NBI images from WLI images for VCE of the esophagus. Three parameters, structural similarity index metric (SSIM), entropy, and peak-signal-to-noise ratio (PSNR), were used to assess the simulated NBI images. Results show the good performance of the NBI image reproduction method with SSIM, entropy difference, and PSNR values of 93.215%, 4.360, and 28.064 dB, respectively.

## 1. Introduction

Video capsule endoscopy (VCE) has been identified as a prospective substitute for traditional endoscopy in recent times [1], owing to the noninvasive nature of the device and its comparatively diminutive size [2,3,4]. The VCE device is designed to include a camera, a miniature lighting system, and an electronic box within its compact form factor, which is comparable in size to a tablet [5,6,7]. Despite its small size, VCE has the ability to generate high-quality images at a significantly higher frame rate [8,9]. Despite the numerous advantages of VCE, a significant drawback of this technology is its lack of narrow band imaging (NBI) functionality, which is commonly present in conventional endoscopes [10]. VCE relies solely on WLI, limiting its ability to detect subtle vascular patterns and mucosal details characteristic of NBI. Furthermore, VCE is prone to several other limitations such as its inability to perform therapeutic interventions, potential for capsule retention in cases of strictures, and challenges in localizing lesions precisely [11]. The exclusion of NBI from VCE means that subtle lesions, which may have crucial clinical implications, could be missed. Furthermore, VCE is a passive imaging modality incapable of therapeutic interventions or biopsies during the procedure, limiting its diagnostic scope and therapeutic utility.

NBI is a modality of medical imaging that employs a specialized filter to selectively permit the transmission of a specific wavelength of light [12,13]. The aforementioned wavelengths have the ability to enhance the contrast, thereby accentuating the features of the mucosal layer [14,15]. Typically, the central wavelength of NBI is either 415 or 540 nm, which correspond to the blue and green spectral bands, respectively [16,17]. The absorption of light by hemoglobin in the bloodstream is heightened at these particular wavelengths to provide enhanced precision, resulting in a darkened appearance of the blood vessels [18,19]. Consequently, the distinguishing features of the mucosa can be readily discerned from the neighboring milieu [20]. NBI bands are predominantly utilized in endoscopy for diagnosing diverse types of cancer [21].

The use of NBI In endoscopy is based on the principle that certain wavelengths of light can enhance the visualization of blood vessels and mucosal patterns in the gastrointestinal tract. Typically, NBI employs either a central wavelength of 415 nm (blue) or 540 nm (green) [22]. These specific wavelengths are chosen because they coincide with the absorption peaks of hemoglobin, the protein responsible for transporting oxygen in blood. Hemoglobin has two primary forms: oxygenated (oxyhemoglobin) and deoxygenated (deoxyhemoglobin). Oxyhemoglobin predominantly absorbs light in the blue spectrum, around 415 nm, whereas deoxyhemoglobin absorbs light in the green spectrum, around 540 nm [23]. When NBI light is directed onto the mucosal surface, it is preferentially absorbed by hemoglobin in blood vessels. This selective absorption results in a darkened appearance of blood vessels against a lighter background, greatly enhancing their visibility during endoscopic examination. This enhanced contrast and precision provided by NBI have proven invaluable in the detection of subtle mucosal changes, such as early neoplastic lesions and vascular patterns indicative of gastrointestinal pathology [24]. Consequently, NBI has become an essential tool in the early diagnosis and surveillance of gastrointestinal diseases.

Specific cancers, such as esophageal EC, which does not have any particular biomarker in early stages, can be diagnosed early with the use of NBI [21,25]. Many previous studies have proven that the accuracy, sensitivity, and specificity of early detection of various types of cancer increased significantly with the use of NBI [26,27,28,29,30,31]. Tsai et al. used single-shot detector and hyperspectral imaging to detect early EC and revealed that the overall accuracy of the NBI images was 91% and the RGB image’s accuracy was only 88% [32]. Lee et al. examined EC by using the conventional white light imaging (WLI) and NBI, and concluded that NBI was much more effective than the conventional WLI in detecting dysplasia [33]. Yosidha et al. demonstrated that the NBI system improved the accuracy of magnifying endoscopy [34]. Hence, the use of NBI is critical in VCE to detect early EC.

Therefore, in this study, the usability of NBI images simulated from decorrelated color spaces for VCE imaging of the esophagus was evaluated. The simulated NBI images were compared with three distinct parameters: PSNR, SSIM, and entropy. The narrow band conversion technology and the dataset employed in this study were discussed in depth. Subsequently, the outcomes obtained from the image comparison using various parameters were elucidated. Finally, a summary of the findings, the potential avenues for future research, and the limitations of this study were provided.

## 2. Materials and Methods

### 2.1. Dataset

Obtaining the necessary dataset for identifying and categorizing the esophagus can often be a challenging undertaking [35]. Moreover, a huge amount of pertinent information can be found in the VCE of the esophagus. In the present study, a series of esophageal images was obtained from two collaborating hospitals. The dataset consisted of a comprehensive collection of 3415 WLI VCE images of the esophagus. WLI images were obtained using VCE (InsightEyes EGD System, Insight Medical Solutions Inc., Hsinchu, Taiwan). VCE images of 640 × 480 pixels in dimension were acquired from Taipei Veterans General Hospital. A supplementary set of 2000 WLI images obtained via a conventional endoscope (CV-290, Olympus, Shinjuku, Tokyo, Japan) was used for analysis. The dataset containing Olympus images was obtained from Chung-Ho Memorial Hospital at Kaohsiung Medical University. The dimension of these images was 640 × 480 pixels.

### 2.2. NBI

The lack of NBI functionality is one of the most significant drawbacks associated with the use of VCE. Increasing the size of the VCE device is not possible because of the convenience factor. Therefore, the NBI features can be added only after the processing is completed. Given that NBI performs far more effectively with computer-aided detection machine learning techniques than WLI, this step is essential for early EC detection. Thus, in this study, a color space that replicated the NBI image was chosen to have decorrelated axes because it has been shown to be an effective tool for manipulating color images. The method developed by Reinhard et al. was applied [36]. Simply imposing the mean and standard deviation (SD) over the data points is a straightforward operation that, when combined with credible input images, results in the production of convincing output images. Only the mean and standard deviation in tandem with any of the three dimensions were needed in this investigation. Therefore, these metrics were calculated for the original image and the target image. A notable detail is that the average and standard deviation for each axis in one space were determined on an individual basis. First, a method that should be considered reasonable for converting RGB signals to l was demonstrated, the perception-based color space developed by Ruderman et al. [37] Considering l is a transform of LMS cone space, the image was converted to LMS space using the LMS transform in two steps. First, the RGB tristimulus values were converted to XYZ ones. By using the standard matrix provided by the International Telecommunications Union, a vector that can be applied to multiply the columns was obtained, resulting in the RGB-to-XYZ conversion. The image was converted for it to be in LMS space using the traditional conversion matrix, as shown in Equation (1).

(1) $$\left(\begin{array}{c} L\\ M\\ S \end{array}\right)=\left(\begin{array}{ccc} 0.3811 & 0.5783 & 0.0402\\ 0.1967 & 0.7244 & 0.0782\\ 0.0241 & 0.1288 & 0.8444 \end{array}\right)\cdot \left(\begin{array}{c} R\\ G\\ B \end{array}\right)$$

The information in this color space exhibited a significant amount of skew, which was reduced to a significant degree after transforming the data to the logarithmic space, as shown in Equation (2).

(2) L=logLM=logMS=logS

First, the data points were taken, and the mean was subtracted from them. Then, the data points that made up the synthetic image were adjusted using the factors determined by the standard deviations of each of the individual data points, as shown in Equation (3).

(3) $$l^{\prime }=\frac{\sigma_{t}^{l}}{\sigma_{s}^{l}}l^{*}\;\alpha^{\prime }=\frac{\sigma_{t}^{\alpha }}{\sigma_{s}^{\alpha }}\alpha^{*}\;\beta^{\prime }=\frac{\sigma_{t}^{\beta }}{\sigma_{s}^{\beta }}\beta^{*}$$

Choosing a source image and a target image that do not go together very well was possible because this investigation aimed to copy the appearance of one image onto another. The compositional similarities between the images can determine the quality of the final product. For instance, if the synthetic image has a significant amount of grass, whereas the photograph has a great amount of sky, then the transformation of statistics could be assumed to be unsuccessful.

### 2.3. Parameters for Comparision

#### 2.3.1. SSIM

SSIM is a widely recognized quality measurement that is utilized in the process of determining how similar two images are to one another [38]. It was introduced by Wang et al., and it has been proposed to be associated with the human visual system’s quality perception [39]. SSIM is developed by predicting every image distortion as an amalgamation of three factors, loss of correlation, contrast distortion, and luminance distortion, rather than using traditional error summation methods [40,41]. SSIM is used as a measure to compare the similarity between WLI Images and NBI images [42]. SSIM metric has gained significant popularity in the field of digital image analysis due to its ease of use, widespread application, and established validity through rigorous testing [43,44]. SSIM values usually range between 0 and 100%, and SSIM values of more than 90% are considered better results [45].

#### 2.3.2. Entropy

The second criterion employed to assess the algorithm formulated in this investigation was entropy [46]. The calculation of entropy was similar to that of SSIM. The entropy discrepancy between the WLI images acquired through the Olympus endoscope and the simulated NBI images was compared. Entropy can be utilized in image processing for the purpose of texture classification. A specific texture may exhibit a distinct entropy value because certain patterns tend to recur in a relatively consistent manner [47]. Within the framework of the paper, low entropy is indicative of reduced disorder and diminished variance among the constituent elements. Thus, reducing the entropy results in an improved reproduction of the image. The entropy disparity between the WLI images acquired through VCE and the simulated NBI images was compared.

#### 2.3.3. PSNR

PSNR is a byte-by-byte comparison of the quality of two images [48,49]. It is one of the simplest methods to compare the source and the reproduced image [50]. PSNR is analyzed similar to SSIM. The corresponding WLI images from VCE and the Olympus endoscope were separately compared with the simulated NBI images. PSRN values range between 20 and 60 dB, and the higher the value is, the better the result. For an 8-bit data representation, the accepted PSNR value is about 25 dB [51].

## 3. Results

In this study, three important parameters were considered for analyzing the effectiveness of the algorithm: PSNR, SSIM, and entropy. Comparing the results of these parameters can help understand the limitations and advantages of the proposed method.

### 3.1. SSIM

**Figure 1:**
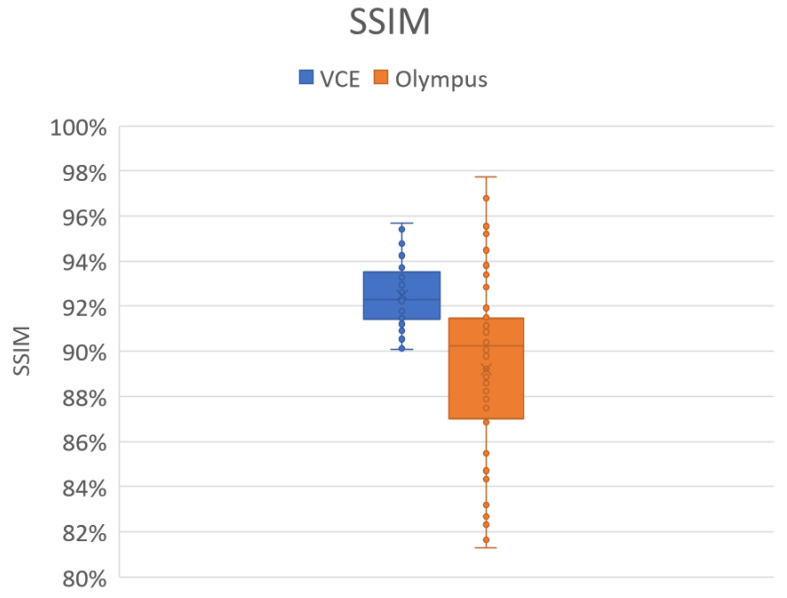
SSIM metric of 50 random Olympus and VCE images.

Figure 1 shows the results of the SSIM analysis. The SSIM value lies in the range of 0 to 100%, with a higher value indicating a better result. In this study, 50 random WLI images and their corresponding simulated NBI images were compared to compare the SSIM between the WLI images and the simulated NBI images. The average SSIM values for the Olympus and VCE images were 90% and 92.49%, respectively. However, out of the 50 images, 21 VCE images had a high SSIM of more than 93%. Out of the 50 randomly selected images, 19 had an SSIM of 91%, and only nine images had less than 84% (Supplementary Table S3). These findings revealed that the average SSIM decreased because of these nine images. These images were either blurred, had too much light reflected on them, or had some flare. Therefore, the SSIM value decreased below 91%. If the dataset was filtered and had a clearer WLI, then the NBI reproduction of the images could be better. Similar to the Olympus images, the eight WLI images in VCE had considerable reflection that made the SSIM value less than 90%. However, in all the different conditions, the SSIM values did not reduce to below 90% in VCE. Therefore, regardless of the errors present in the image, the NBI reproduction can be profound.

### 3.2. Entropy

**Figure 2:**
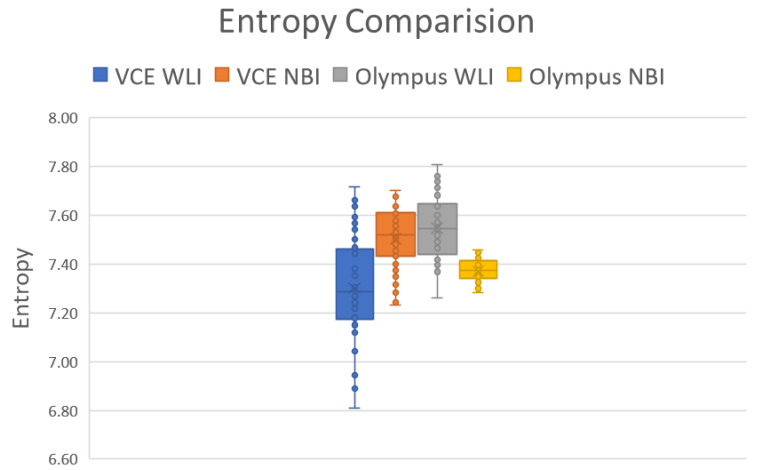
Entropy of 50 randomly chosen Olympus and VCE images.

The entropy difference between the Olympus endoscope and VCE is illustrated in Figure 2. The average entropy difference between the WLI and simulated NBI images in VCE was around 2.6942%, and average entropy difference between the WLI and simulated images from the Olympus endoscope was 2.3457% (Supplementary Table S2). The results showed that the entropy in the Olympus endoscope and VCE followed a similar pattern: when the entropy increased in the WLI images, the entropy of the NBI images also increased, and vice versa. In VCE and the Olympus endoscope, the entropy in the NBI images increased because of three images (image numbers 20, 25, and 46). This finding can be attributed to the excessive reflection seen in the WLI image, indicating the successful utilization of the algorithm in this study. The differences in entropy between images provide insight into the levels of randomness or disorder present in the pixel intensity values within each image. Entropy differences between the same images can indicate variations in information content or randomness within the images. Higher entropy differences suggest greater dissimilarity between the images, potentially due to noise, compression artifacts, or alterations. Conversely, lower entropy differences imply more similarity or consistency between the images, indicating minimal changes or distortions. Analyzing these differences can be useful in quality control, image restoration, or change detection applications, helping identify and quantify the extent of image alterations or discrepancies and ultimately aiding in image analysis and processing tasks.

### 3.3. PSNR

**Figure 3:**
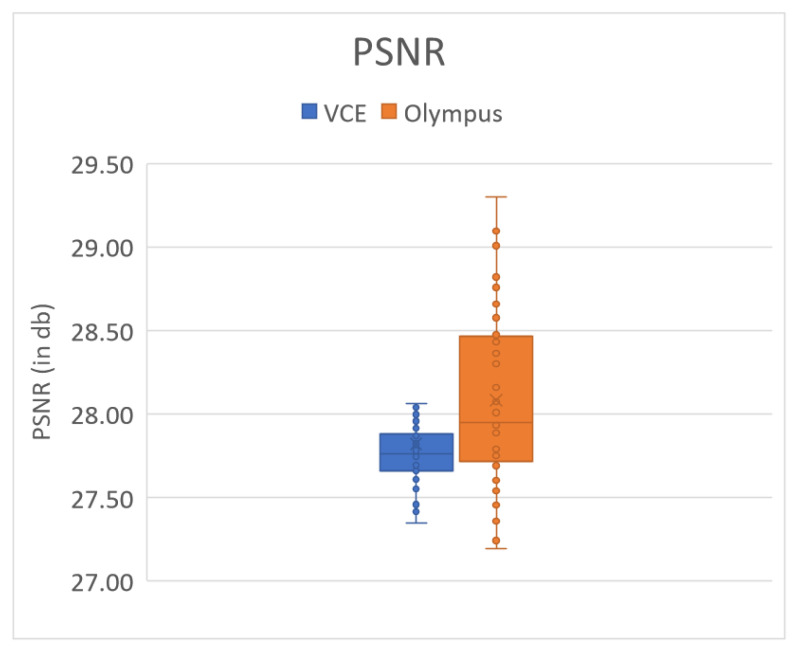
PSNR comparison of 50 randomly chosen Olympus and VCE images.

Figure 3 shows the PSRN value of the 50 randomly chosen VCE and Olympus endoscopic images. The average PSNR values of the VCE and Olympus endoscopic images were 27.8212 and 28.0813 dB, respectively (Supplementary Table S1). The results of SSIM, entropy, and PSNR showed that the proposed algorithm performed better.

## 4. Discussion

**Figure 4:**
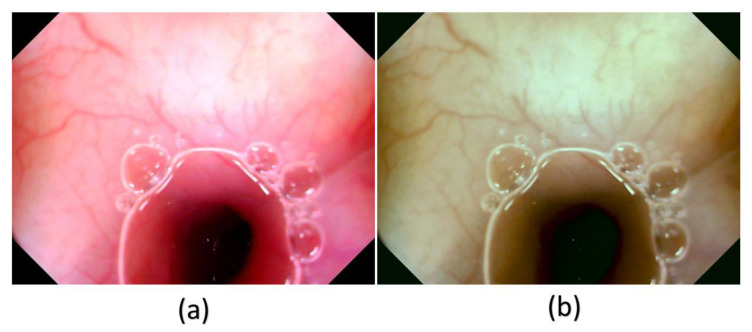
Comparison between WLI and simulated NBI images of VCE. (**a**) WLI image from VCE, (**b**) simulated NBI image from the NBI conversion algorithm.

**Figure 5:**
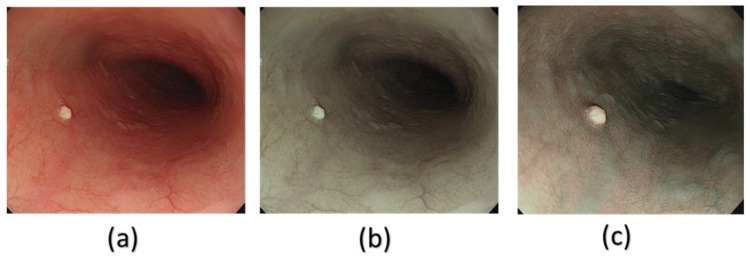
Comparison among the (**a**) WLI images, (**b**) simulated NBI images, and (**c**) real NBI images from the Olympus endoscope.

In this study, a decolored axis color-matching function was used to simulate NBI images from the VCE WLI images by using the NBI image from an Olympus endoscope as a reference. Given that VCE is more preferred than the traditional endoscopy, incorporating NBI capability onto VCE is an important requisite because VCE does not currently have NBI functionality. Such functionality has been proven to be more effective in detecting early cancer cells, specifically EC, which does not have any early biomarkers. This incorporation could increase the 5-year survival rate of EC drastically. The results of this study showed that the reproduced NBI images had better comparison metric values. First, the average SSIM values of the Olympus and VCE endoscopic images were 89.1995% and 92.4919%, respectively. Second, the average entropy values of the randomly chosen WLI images and their corresponding NBI images in VCE and the Olympus endoscope were 2.6942% and 2.3457%, respectively. Finally, the PSNRs of the WLI images and their corresponding NBI images in VCE and the Olympus endoscope were 27.8212 and 28.0813 dB, respectively. The future scope of this study is to test the reproduced NBI images with a YOLOv5 deep learning model with a dataset of EC to detect and classify cancers on the basis of stage severity. Then, the same model could be used to compare the accuracy, sensitivity, and specificity of WLI of those NBI. However, one of the limitations of this method is that it did not consider the lighting spectrum of the WLI and NBI images. Therefore, if the WLI image is blurred or has light reflections on it, the NBI image could not be a perfect simulation. The simulated NBI image with the corresponding WLI of the VCE endoscope is shown in Figure 4 (Supplementary Figure S1 shows 50 randomly chosen images of WLI in VCE, and Figure S2 shows 50 randomly chosen images of the simulated NBI in VCE). The simulated NBI image with the corresponding WLI and a similar NBI image from the Olympus endoscope are shown in Figure 5 (Supplementary Figure S3 shows six randomly chosen images of WLI in VCE, and Figures S3 and S4 shows another 12 randomly chosen images of simulated NBI in VCE).

## 5. Conclusions

In this study, a decolored axis color-matching function was implemented to simulate NBI images from VCE WLI images by making use of the NBI image obtained from an Olympus endoscope as a reference. VCE does not currently have NBI functionality, which has been proven to be more effective in detecting early cancer cells, particularly early EC, which does not have any early biomarkers. Given that VCE is more preferred than the traditional endoscopy, incorporating NBI capability into VCE is an important requisite that could result in a significant increase in the 5-year survival rate of patients with EC. The results showed that the reproduced NBI images had higher comparison metric values. The images from the Olympus endoscope and VCE had average SSIM values of 98.415% and 93.215%, respectively. The random WLI images and their corresponding NBI images obtained from VCE and the Olympus endoscopes showed average entropy values of 3.45% and 4.36%, respectively. The PSNRs of the WLI images with their corresponding NBI images in VCE and the Olympus endoscope were 28.06 and 28.15%, respectively.

## Data Availability

The data presented in this study are available in this article upon request to the corresponding author.

## Supplementary Materials

The following supporting information can be downloaded at https://www.mdpi.com/article/10.3390/cancers15194715/s1, Table S1: Results of PSNR comparison of each image in Olympus and VCE; Table S2: Results of Entropy comparison of each image in Olympus and VCE; Table S3: Results of SSIM comparison of each image in Olympus and VCE; Figure S1: 50 Randomly chosen images of WLI in VCE; Figure S2: 50 Randomly chosen images of NBI in VCE; Figure S3: 6 Randomly choses WLI images, simulated NBI images and a similar original NBI in Olympus endoscope. (a) Olympus WLI images. (b) simulated NBI image and (c) a similar NBI image from Olympus; Figure S4: 6 Randomly choses WLI images, simulated NBI images and a similar original NBI in Olympus endoscope. (a) Olympus WLI images. (b) simulated NBI image and (c) a similar NBI image from Olympus.
