# Supplementary material for: Assessment of Narrow Band Imaging Algorithm for Video Capsule Endoscopy Based on Decorrelated Color Space for Esophageal Cancer
Source: Cancers (Basel). 2023 Sep 25;15(19):4715. doi: 10.3390/cancers15194715 (PMC10571786; doi:10.3390/cancers15194715)
Supplement: Supplementary file 1 [file cancers-15-04715-s001.zip › cancers-2560633-supplementary.pdf]

cancers

MDPI

Article

Assessment of Narrow Band Imaging Algorithm for Video Capsule Endoscopy Based on Decorrelated Color Space for Esophageal Cancer

Kai-Yao Yang [1,†], Yu-Jen Fang [2,3,†], Riya Karmakar [4], Arvind Mukundan [4], Yu-Ming Tsao [4], Chien-Wei Huang [1,5,*] and Hsiang-Chen Wang [4,6,7,*]

[1] Department of Medical Material Research, Kaohsiung Armed Forces General Hospital, 2, Zhongzheng 1st.Rd., Lingya District, Kaohsiung City 80284, Taiwan; yangkaiyao@gmail.com
[2] Department of Internal Medicine, National Taiwan University Hospital, Yun-Lin Branch, No. 579, Sec. 2, Yunlin Rd., Dou-Liu 64041, Taiwan; toby851072@gmail.com
[3] Department of Internal Medicine, National Taiwan University College, No. 1 Jen Ai Rd. Sec. 1, Taipei 10051, Taiwan
[4] Department of Mechanical Engineering, National Chung Cheng University, 168, University Rd., Min Hsiung, Chia Yi 62102, Taiwan; karmakarriya345@gmail.com (R.K.); d09420003@ccu.edu.tw (A.M.); d09420002@ccu.edu.tw (Y.-M.T.)
[5] Department of Nursing, Tajen University, 20, Weixin Rd., Yanpu Township, Pingtung County 90741, Taiwan
[6] Department of Medical Research, Dalin Tzu Chi Hospital, Buddhist Tzu Chi Medical Foundation, No. 2, Minsheng Road, Dalin, Chiayi 62247, Taiwan
[7] Hitspectra Intelligent Technology Co., Ltd., 4F, No.2, Fuxing 4th Rd., Qianzhen District, Kaohsiung 80661, Taiwan
* Correspondence: forevershiningfy@yahoo.com.tw (C.-W.H.); hcwang@ccu.edu.tw (H.-C.W.)
† These authors contributed equally to this work.

Citation: Yang, K.-Y.; Fang, Y.-J.; Karmakar, R.; Mukundan, A.; Tsao, Y.-M.; Huang, C.-W.; Wang, H.-C. Assessment of Narrow Band Imaging Algorithm for Video Capsule Endoscopy Based on Decorrelated Color Space for Esophageal Cancer. *Cancers* **2023**, *15*, 4715. https://doi.org/10.3390/cancers15194715

Academic Editors: Hajime Isomoto, Michalis V. Karamouzis and Dimitrios Schizas

Received: 31 July 2023
Revised: 15 September 2023
Accepted: 24 September 2023
Published: 25 September 2023

Copyright: © 2023 by the authors. Licensee MDPI, Basel, Switzerland. This article is an open access article distributed under the terms and conditions of the Creative Commons Attribution (CC BY) license (https://creativecommons.org/licenses/by/4.0/).

1. Equations for Image Comparisons

The entropy of an image is defined as follows:

$$-\sum_{i=0}^{n-1} p_i \log_b p_i ,$$

where *n* is the number of gray levels (256 for 8-bit images), *pi* is the probability of a pixel having gray level *i*, and *b* is the base of the logarithm function. The entropy of an image is rather different from the entropy feature extracted from the gray-level co-occurrence matrix of an image.

Structural similarity index measure (SSIM) is a method for predicting the perceived quality of digital television and cinematic pictures, as well as other kinds of digital images and videos. It is used for measuring the similarity between two images. It can be represented by the following equation:

$$\mathrm{SSIM}(x,y) = \frac{(2\mu_x\mu_y + c_1)(2\sigma_{xy} + c_2)}{(\mu_x^2 + \mu_y^2 + c_1)(\sigma_x^2 + \sigma_y^2 + c_2)},$$

where μ_x is the pixel sample mean of x, μ_y is the pixel sample mean of y, C_1 and C_2 are variables to stabilize the division with weak denominator, $K_1 = 0.01$ and $K_2 = 0.03$, σ_{xy} refers to the covariance of x and y, L denotes the dynamic range of pixel values, σ_x^2 is the covariance of x, and σ_y^2 is the covariance of y.

PSNR is most easily defined via the mean squared error (*MSE*). Given a noise-free $m \times n$ monochrome image I and its noisy approximation K, *MSE* is defined as follows:

$$MSE = \frac{1}{mn}\sum_{i=0}^{m-1}\sum_{j=0}^{n-1}[I(i,j) - K(i,j)]^2.$$

PSNR (in dB) is defined as follows:

$$\begin{aligned} PSNR &= 10 \cdot \log_{10}\left(\frac{MAX_I^2}{MSE}\right) \\ &= 20 \cdot \log_{10}\left(\frac{MAX_I}{\sqrt{MSE}}\right) \\ &= 20 \cdot \log_{10}(MAX_I) - 10 \cdot \log_{10}(MSE). \end{aligned}$$

Here, MAX_I is the maximum possible pixel value of the image. When the pixels are represented using 8 bits per sample, the MAX_I is 255. In general, when samples are represented using linear PCM with B bits per sample, the MAX_I is $2^B - 1$.

2. Results of Image Comparison

Table S1. Results of PSNR comparison of each image in Olympus and VCE

PSNR Comparision		
# image	Olympus	VCE
1	28.3033	28.4234
2	27.5851	27.4912
3	28.6736	28.012
4	27.656	29.008
5	27.6027	29.0184
6	27.782	28.8618
7	28.4473	28.0175
8	27.8109	28.5319
9	27.5827	28.3681
10	27.5073	27.9671
11	27.6241	27.8602
12	27.646	27.8653
13	29.6058	27.7043
14	28.028	27.9716
15	29.7888	27.6491
16	29.2247	27.6757
17	27.7772	27.5595
18	28.4386	27.7358
19	27.7769	27.8191
20	28.1393	27.7566
Average	28.15003	28.06484

Table S2. Results of Entropy comparison of each image in Olympus and VCE

Entropy Comparision				
# Images	Olympus		Vce	
	WLI	NBI	WLI	NBI
1	7.4692	7.17155	6.43501	6.950931
2	7.696849	7.55283	6.55779	6.95735
3	7.64243	7.37639	6.43802	6.88498
4	7.58609	7.34965	6.47801	6.93397
5	7.46084	7.08171	6.52072	7.00907
6	7.51781	7.33392	6.66476	7.01961
7	7.34168	6.84817	6.49488	7.1053
8	7.41681	7.10808	6.5661	7.19722
9	7.46113	7.24866	6.53207	7.17257
10	7.50348	7.24452	6.86103	7.19379
11	7.41662	7.18721	6.99237	7.26331
12	7.42663	7.16701	7.06938	7.27016
13	7.52686	7.42154	7.10936	7.27413
14	7.74991	7.62265	7.10993	7.25245
15	7.63398	7.35181	7.03728	7.18075
16	7.48843	7.31261	7.04451	7.1778
17	7.53016	7.21741	7.07349	7.19443
18	7.44276	7.20028	7.10469	7.16334
19	7.52783	7.19222	7.13437	7.19183
20	7.46343	7.29484	7.14958	7.19768
Average	7.515147	7.264154	6.818668	7.129534
Difference	3.455%		4.559%	

Table S3. Results of SSIM comparison of each image in Olympus and VCE

SSIM Comparision		
# Number	Olympus	VCE
1	98.37	92.65
2	96.91	92.86
3	98.6	92.49
4	99.7	92.23
5	99.56	91.74
6	99	92.15
7	99.64	91.17
8	99.55	92.12
9	99.66	92.1
10	99.35	93.42
11	96.11	94.09
12	99.3	94.2
13	97.12	94.24
14	99.45	94.21
15	98.3	94.12
16	99.32	94.07
17	97.42	94.07
18	96.11	94.1
19	96.72	94.16
20	99	94.2
Average	98.4595	93.2195

S3. Image examples of NBI conversion algorithm

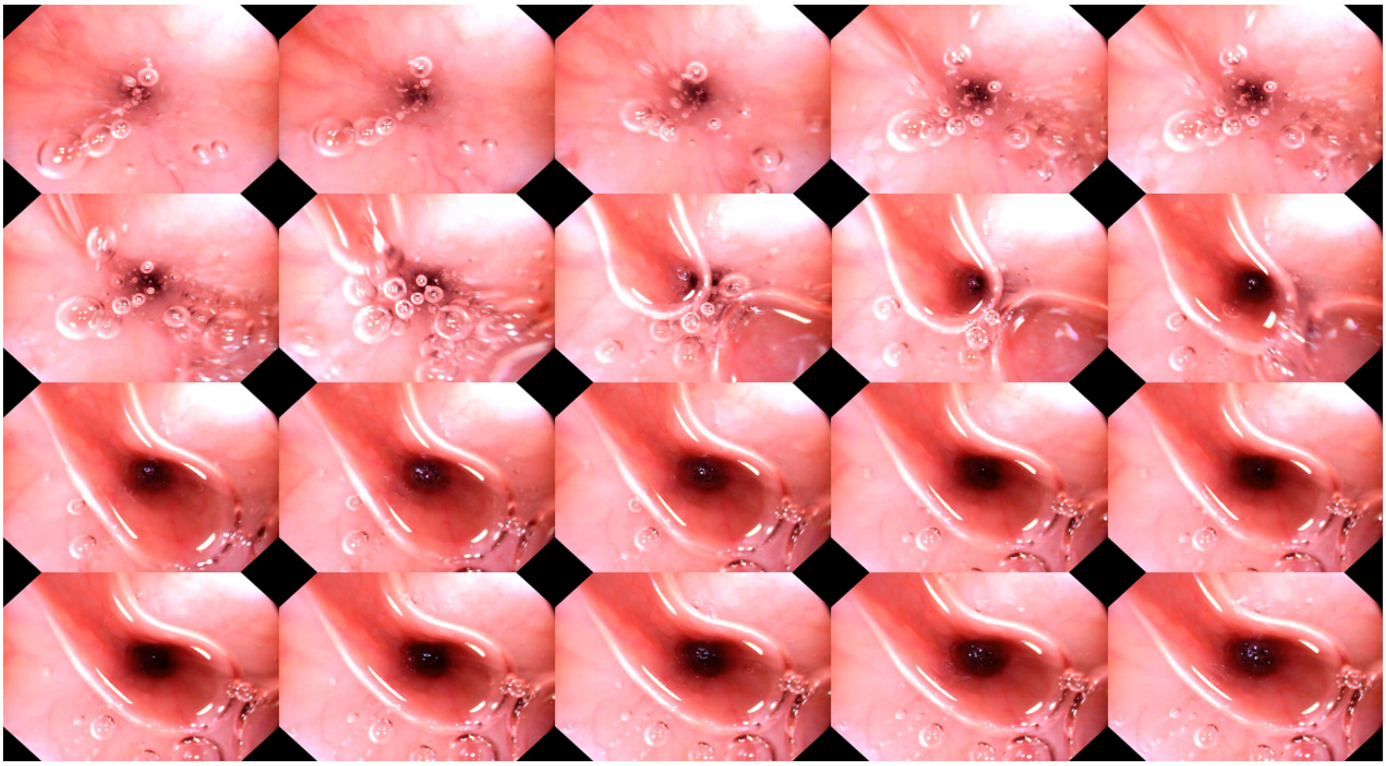

Figure S1. 20 Randomly chosen images of WLI in VCE

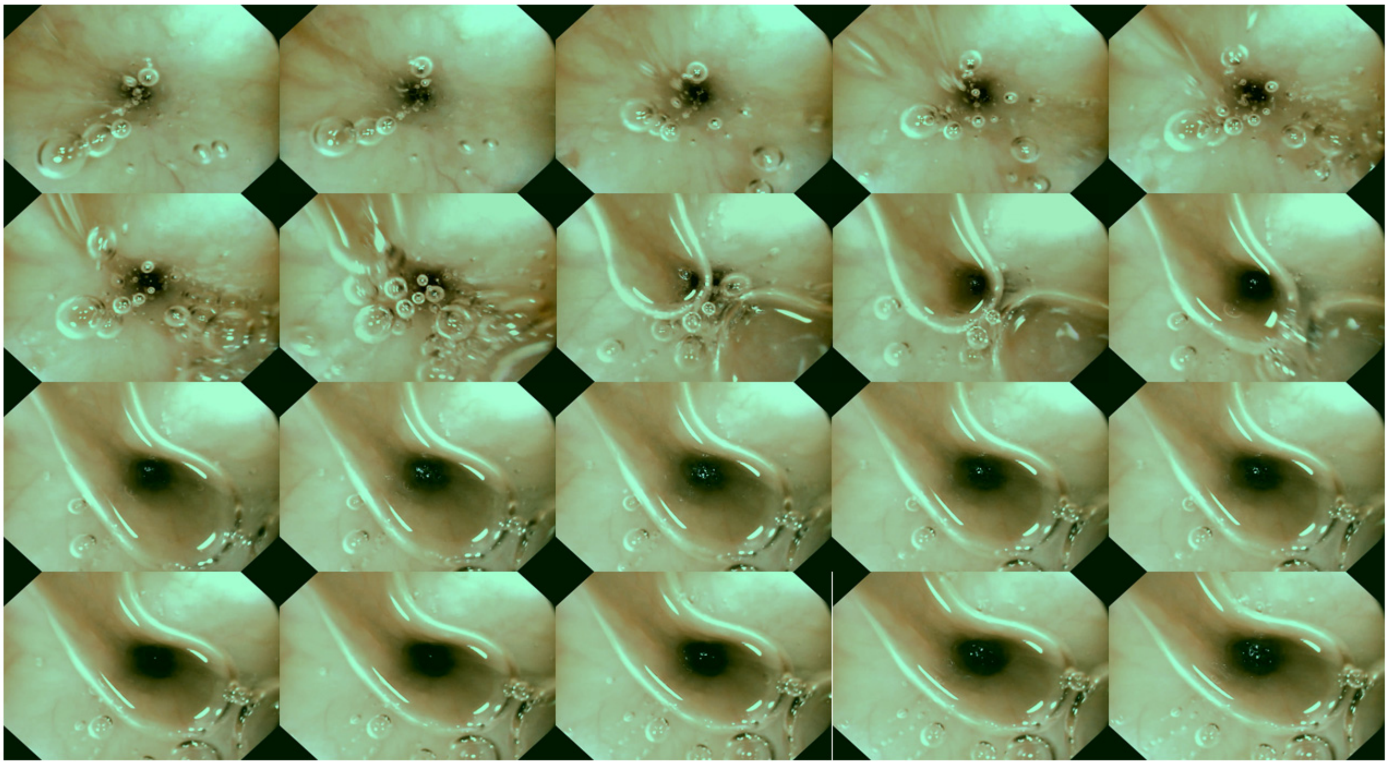

Figure S2. Twenty randomly chosen images of NBI in VCE

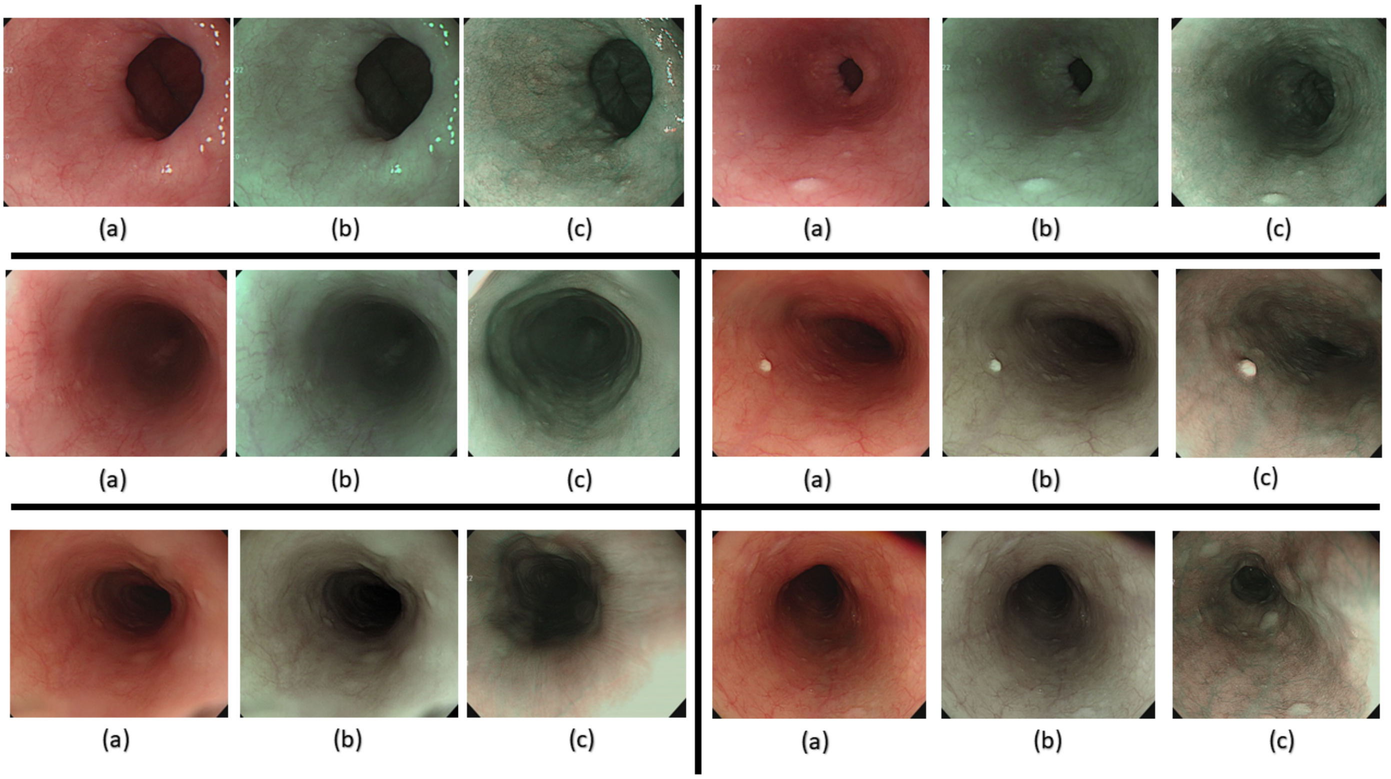

Figure S3. Six randomly chosen (a) WLI images, simulated NBI images, and a similar original NBI from Olympus endoscope

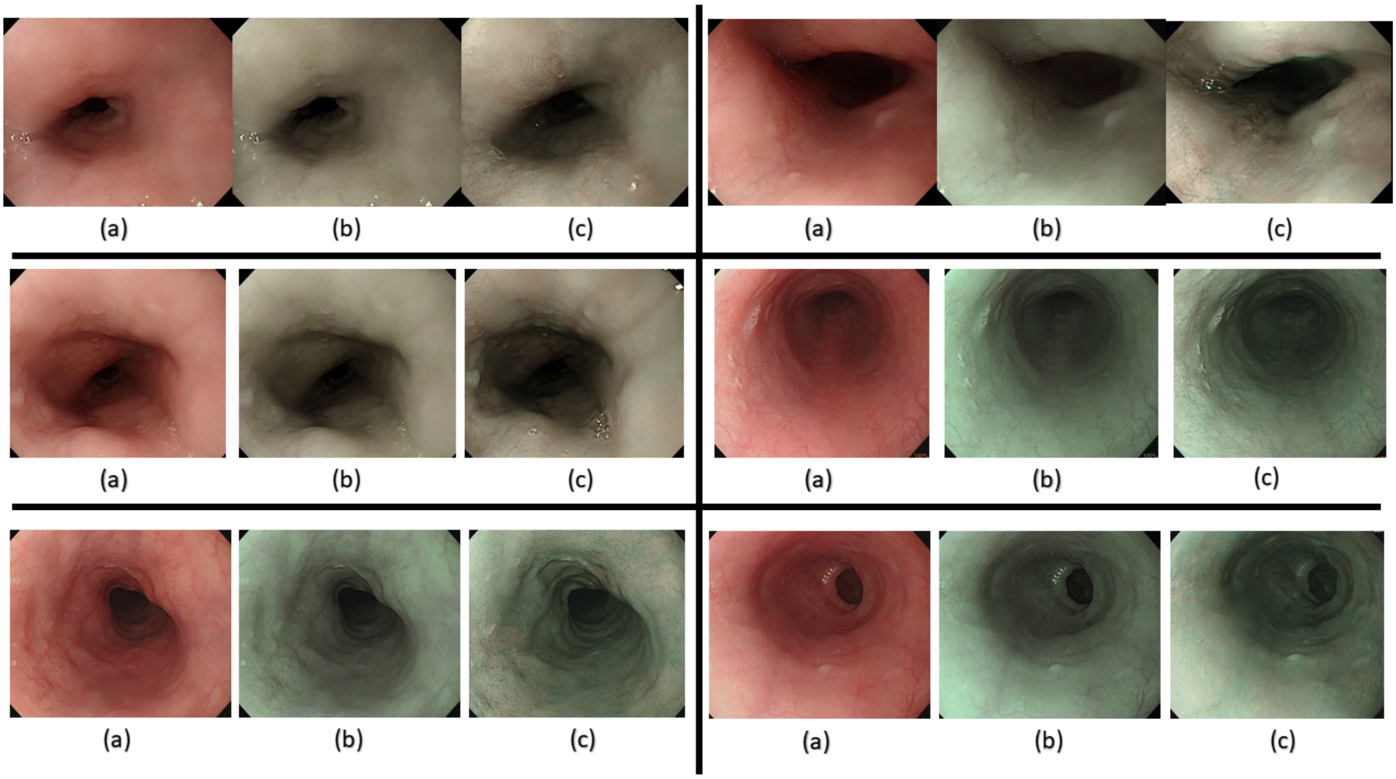

Figure S4. Six randomly chosen (a) WLI images, (b) simulated NBI images, and (c) similar original NBI images from Olympus endoscope

Disclaimer/Publisher's Note: The statements, opinions and data contained in all publications are solely those of the individual author(s) and contributor(s) and not of MDPI and/or the editor(s). MDPI and/or the editor(s) disclaim responsibility for any injury to people or property resulting from any ideas, methods, instructions or products referred to in the content.
